# Horizontal gene transfer as a mechanism for the promiscuous acquisition of distinct classes of IRES by avian caliciviruses

**DOI:** 10.1093/nar/gkab1243

**Published:** 2021-12-20

**Authors:** Yani Arhab, Anna Miścicka, Tatyana V Pestova, Christopher U T Hellen

**Affiliations:** Department of Cell Biology, SUNY Downstate Health Sciences University, Brooklyn NY 11203, USA; Department of Cell Biology, SUNY Downstate Health Sciences University, Brooklyn NY 11203, USA; Department of Cell Biology, SUNY Downstate Health Sciences University, Brooklyn NY 11203, USA; Department of Cell Biology, SUNY Downstate Health Sciences University, Brooklyn NY 11203, USA

## Abstract

In contrast to members of *Picornaviridae* which have long 5′-untranslated regions (5′UTRs) containing internal ribosomal entry sites (IRESs) that form five distinct classes, members of *Caliciviridae* typically have short 5′UTRs and initiation of translation on them is mediated by interaction of the viral 5′-terminal genome-linked protein (VPg) with subunits of eIF4F rather than by an IRES. The recent description of calicivirus genomes with 500–900nt long 5′UTRs was therefore unexpected and prompted us to examine them in detail. Sequence analysis and structural modelling of the atypically long 5′UTRs of *Caliciviridae* sp. isolate yc-13 and six other caliciviruses suggested that they contain picornavirus-like type 2 IRESs, whereas ruddy turnstone calicivirus (RTCV) and *Caliciviridae* sp. isolate hwf182cal1 calicivirus contain type 4 and type 5 IRESs, respectively. The suggestion that initiation on RTCV mRNA occurs by the type 4 IRES mechanism was confirmed experimentally using *in vitro* reconstitution. The high sequence identity between identified calicivirus IRESs and specific picornavirus IRESs suggests a common evolutionary origin. These calicivirus IRESs occur in a single phylogenetic branch of *Caliciviridae* and were likely acquired by horizontal gene transfer.

## INTRODUCTION

Genetic variation in viral genomes arises from point mutation and recombination. The former allows for gradual searching through an evolutionary fitness landscape, whereas recombination is associated with large shifts that may create beneficial genetic diversity or disrupt favorable combinations of co-adapted alleles (1,2). Recombination in RNA virus genomes has been associated with increased virulence, altered host range and the emergence of viruses (3–7). It can occur by a replicative mechanism, in which the replication complex transfers from one template to another, or by a non-replicative mechanism in which genomes are cleaved and joined in new combinations (8). These processes can result in non-homologous recombination, by joining of fragments of similar genomes at dissimilar locations or of unrelated RNA molecules. The latter leads to horizontal gene transfer (HGT) between unrelated genomes and to the acquisition of genetic information. Analysis of HGT has focused on the transfer of protein-coding regions between viruses and from hosts (9). However, noncoding regions in viral RNA genomes, which have roles in translation, replication and encapsidation, are also heritable entities and just as for coding sequences, their evolution may also involve recombination and HGT between members of the same and even of different virus families (10–15). 5′-Untranslated regions (5′UTRs) are of particular interest because in a number of viral mRNAs, they contain specific elements that allow the viral mRNAs to utilize non-canonical 5′end-independent mechanisms of initiation that are collectively termed ‘internal ribosomal entry’.

The canonical initiation process involves attachment of 43S preinitiation complexes (comprising 40S ribosomal subunits, eIF2-GTP/ Met-tRNA_i_^Met^ ternary complexes and eIFs 3, 1 and 1A) to the capped 5′-terminal region of mRNA and their subsequent scanning to the initiation codon where they stop to form 48S initiation complexes with established codon-anticodon base-pairing. Attachment is mediated by group 4 eIFs: eIF4F (which consists of the RNA helicase eIF4A, the scaffold subunit eIF4G and the cap-binding subunit eIF4E), eIF4A (which also exists in the free form), and eIF4B (which enhances the helicase activity of eIF4A). Group 4 eIFs cooperatively unwind the cap-proximal region allowing attachment of 43S complexes and also assist 43S complexes during scanning. eIFs 1 and 1A monitor the fidelity of initiation codon selection. Establishment of codon-anticodon base-pairing in the 48S complex leads to eIF5-induced hydrolysis of eIF2-bound GTP, eIF5B-mediated joining of a 60S ribosomal subunit and formation of elongation-competent 80S ribosomes (16).

Internal ribosomal entry sites (IRESs) are structured RNA regions that mediate end-independent initiation of translation using a subset of the eukaryotic initiation factors (eIFs) that are required by the canonical initiation process (16). IRESs enable viral mRNAs to be translated during virus-induced shut-off of cellular translation and to evade innate immune responses that repress translation. Viral internal ribosomal entry sites (IRESs) are classified into six major groups, based on common sequence motifs and structure (Table 1). Each group uses a distinct mechanism to assemble ribosomal initiation complexes, but they are all based on non-canonical interactions of the IRES with canonical components of the translation apparatus (16,17).

**Table 1.** Classes of viral IRES

IRES Class	Representative member	Virus family	Structural domains
1	Poliovirus	*Picornaviridae*	II, III, IV, V, VI
2	Encephalomyocarditis virus	*Picornaviridae*	H, I, J, K, L
	Caliciviridae sp. isolate yc-13	*Caliciviridae*	H, I, J, K
3	Hepatovirus A	*Picornaviridae*	IIa/IIb, IIIa/IIIb, IV, V, VI
4	Hepatitis C virus	*Flaviviridae*	II, III, IV
	Teschovirus A	*Picornaviridae*	II, III
	Ruddy turnstone calicivirus A	*Caliciviridae*	II, III
5	Aichivirus A	*Picornaviridae*	I, J, K, L
	Caliciviridae sp. isolate hwf182cal1	*Caliciviridae*	I, J, K, L
6	Cricket paralysis virus	*Dicistroviridae*	1, 2, 3

Initiation on type 1, type 2 and type 5 IRESs, exemplified by poliovirus, encephalomyocarditis virus (EMCV) and Aichivirus (AV) respectively, relies on their specific interaction with the central eIF4A-binding domain of eIF4G (11,18–25). This interaction allows these IRESs to function without eIF4E and the N-terminal region of eIF4G to which it binds, for example in infected cells, when host cell translation is shut off following cleavage of eIF4G by viral proteases into this N-terminal fragment and a C-terminal fragment that binds eIF4A and eIF3. Type 1 and type 2 IRESs are ∼450 nt long and consist of five domains, designated II–VI in type 1 and H–L in type 2 IRESs. Sequence similarities between type 1 and type 2 IRESs are minimal except for a 3′-terminal Yn-Xm-AUG motif, in which a Yn pyrimidine tract (n = 8–10 nt) is separated by a spacer (m = 18–20 nt) from an AUG triplet. Type 5 IRESs are also ∼450 nt long and appear to be chimeric, containing one domain that resembles domain IV of type 1 IRESs, another that resembles domain K of type 2 IRESs, and a Yn-Xm-AUG motif. The AUG of this motif is the initiation codon for the viral polyprotein in type 2 and type 5 IRESs, although initiation can also occur downstream of it in type 2 IRESs, whereas it is sequestered within domain VI in type 1 IRESs and is only weakly active. Translation of the poliovirus polyprotein initiates ∼160 nt downstream of the motif. 48S complex formation on type 2 IRESs requires eIF2, eIF3, the central domain of eIF4G and eIF4A, and is enhanced by eIF4B (18–20); scanning to AUG codons downstream of the Yn-Xm-AUG motif additionally requires eIF1 and eIF1A (26). Initiation on type 1 IRESs requires eIF2, eIF3, eIF4A, eIF4B, the central domain of eIF4G and eIF1A, and scanning beyond the Yn-Xm-AUG motif additionally required eIF1 (25). In addition to canonical eIFs, these IRESs also commonly require specific IRES *trans*-acting factors (ITAFs). Thus the principal ITAF for type 1 IRESs is the poly(C) binding protein 2 whereas Type 2 IRESs require the pyrimidine tract binding protein (PTB).

Whereas the domain organization of type 1, type 2 and type 5 IRESs and their initiation mechanisms are broadly similar, the structures and mechanisms of action of type 4 and type 6 IRESs are fundamentally different from each other and from other classes of IRES. Type 4 IRESs are exemplified by hepatitis C virus (HCV) and classical swine fever virus (CSFV). The mechanism of initiation on type 4 IRESs is based on their direct specific interaction with 40S subunits, which positions the initiation codon in the ribosomal P site so that the 40S/IRES complex can recruit eIF2-GTP/Met-tRNA_i_^Met^ and form a 48S complex without the involvement of group 4 eIFs (28–35). In addition to 40S subunits, type 4 IRESs also specifically interact with eIF3. However, in 40S/IRES/eIF3 complexes, eIF3 is displaced from its ribosomal position in the 43S complex, and instead interacts through its ribosome-binding surface exclusively with the IRES (35). As in the canonical initiation process, subunit joining on type 4 IRESs is mediated by eIF5 and eIF5B (36), but during viral infection and other stress conditions, when active eIF2 levels are reduced, eIF5B can also promote recruitment of Met-tRNA_i_^Met^ independently of eIF2 (37,38). Type 4 IRESs are ∼330nt long and consist of two principal domains: domain II, which is an irregular stem-loop, and domain III, which consists of a basal pseudoknot (PK) and the branching stemloops IIIa - IIIf, several of which contain conserved motifs that are responsible for tertiary interactions within the IRES (30) and for interactions with 18S rRNA of the 40S subunit (28,29). The apical region of domain III of the IRES also interacts with eIF3 (32–35). Type 4 IRESs occur in the *Hepacivirus*, *Pestivirus* and *Pegivirus* genera of *Flaviviridae*, and in over twenty genera of Picornaviridae, including *Teschovirus A* (formerly porcine teschovirus; genus *Teschovirus*) and *Sapelovirus A* (formerly Simian picornavirus 9; genus *Sapelovirus*) (e.g. 10,13,15,39–41). Type 6 IRESs are only ∼190 nt long and consists of two highly structured domains formed by three pseudoknots. They bind directly to the ribosome, and by mimicking the anticodon stem-loop of tRNA base-paired to an mRNA codon, the 3′-terminal pseudoknot enables these IRESs to initiate without the involvement of eIFs or Met-tRNA_i_^Met^ even an initiation codon (16,17).

*Picornaviridae* and *Caliciviridae* are families of viruses in the order *Picornavirales* that have single-stranded, positive-sense RNA genomes. Calicivirus genomic mRNA contain the large open reading frame ORF1 that encodes replicative proteins, followed by one to three additional ORFs that encode capsid proteins, and that are translated from subgenomic mRNA by a process that for ORF3 involves reinitiation (42,43). In contrast to picornaviruses, caliciviruses have short 5′UTRs (44) and initiation of translation on them is mediated by interaction of the viral 5′-terminal genome-linked protein (VPg) with subunits of eIF4F rather than by an IRES (45–47). Consequently, the recent identification of calicivirus genomes with 5′UTRs that are 500–900nt long (48–51) was unexpected and prompted us to examine them in detail. We determined that different avian calicivirus genomes contain type 2, type 4 and type 5 IRESs that were likely acquired from picornaviruses on multiple occasions. These observations provide further evidence for HGT of noncoding RNA elements as a contributory element to viral evolution. Detailed characterization of the mechanism of initiation on the ruddy turnstone calicivirus (RTCV) IRES supported its identification as a type 4 IRES and deepened understanding of the mechanism of initiation on this class of IRES.

## MATERIALS AND METHODS

### Sequences

Sequences were analysed from the following caliciviruses (name followed by Genbank accession number): *Caliciviridae* sp. isolate hwf182cal1 (MT138020.1), *Caliciviridae* sp. isolate xftoti59cal1 (MT138028.1), grey teal calicivirus isolate MW09 (MK204392.1), duck calicivirus isolate MW20 (MH453811.1), pink-eared duck calicivirus I isolate MW23 (MK204416.1), *Caliciviridae* sp. isolate yc-13 (KY312552.1), avocet calicivirus isolate MW21 (MH453804.1), ruddy turnstone calicivirus A isolate MW19 (MH453861.1), Wilkes virus isolate Antarctic11 (MT025075.1), calicivirus chicken/V0021/Bayern/2004 (genus *Bavovirus*) (HQ010042.1), European brown hare syndrome virus (Z69620.1) and rabbit hemorrhagic disease virus (RHDV) FRG (M67473.1) (genus *Lagovirus*), fathead minnow calicivirus (genus *Minovirus*) (KX371097.1), turkey calicivirus isolate L11043 (genus *Nacovirus*) (JQ347522.1), Newbury agent 1 virus (genus *Nebovirus*) (DQ013304.1), Norwalk virus (M87661.2) and murine norovirus 1 (MNV1) clone CW1 (DQ285629.1) (Genus *Norovirus*), Tulane virus (genus *Recovirus*) (EU391643.1), Atlantic salmon calicivirus isolate Nordland/2011 (genus *Salovirus*) (KJ577139.1), sapovirus Hu/Dresden/pJG-Sap01/DE (HM002617.1) (genus *Sapovirus*), St-Valérien calicivirus. isolate pig/AB90/CAN (FJ355928.1) (genus Valovirus), feline calicivirus (FCV) (genus *Vesivirus*) (M86379.1) and FCV strain Urbana (L40021), goose calicivirus isolate H146 (proposed genus ‘*Sanovirus*’) (KY399947.1), duck calicivirus 2 strain DuCV-2_B6 (MN175552.1), goose calicivirus strain N (KJ473715.1), and turkey calicivirus isolate L11043 (JQ347522.1).

Sequences were analysed from the following picornaviruses (name followed by Genbank accession number): Anativirus A (AnV; formerly duck picornavirus TW90A) (AY563023.1), Avisivirus A1 strain turkey/M176-TuASV/2011/HUN (KC465954.1), chicken gallivirus 1 isolate 518C (KF979337.1), Gallivirus A1 strain turkey/M176/2011/HUN (JQ691613.1), Sicinivirus sp. strain RS/BR/2015/5R (MG846487.1), Oscivirus A2 thrush/Hong Kong/10878/2006 (GU182410.1), *Passerivirus* sp. strain waxbill/DB01/HUN/2014 (MF977321.1), Phacovirus Pf-CHK1/PhV (KT880670.1), avocet picornavirus isolate MW13 (MH453807.1), and quail picornavirus QPV1/HUN/2010 (JN674502.1).

### Plasmids

Expression vectors for His_6_-tagged eIF1 and eIF1A (52), eIF4A^R362Q^ (53), eIF5 (54) and the transcription vectors for dicistronic HCV IRES-containing mRNA (pXL.HCV(40–373).NS’ (55), here renamed DC HCV) and tRNA_i_^Met^ (56) have been described. pUC57-T7-EMCV(373–1656) was made by GenScript (Piscataway, NJ) by inserting EMCV nt. 373–1656 (Genbank M81861.1) with 3′-terminal EcoRV, XhoI and EcoRI sites downstream of a T7 promoter in pUC57.

The monocistronic transcription vectors pUC57-T7-Stem-[RTCV nt1-1488], pUC57-T7-Stem-[RTCV nt210-1488], pUC57-T7-Stem-[RTCV nt1-1488](GGG_451-453_-CCC) and pUC57-T7-Stem-[RTCV nt210-1488](GGG_451-453_CCC) were prepared (Genscript) by inserting DNA comprising a HindIII site, a T7 promoter, a hairpin (5′-GGGCCCGACCCGGTGACGGGTCGGGCCC-3′) (Δ*G* = −32.40 kcal/mol) and RTCV nt.1–1488 or nt. 210–1488, with or without GGG_451–453_-CCC substitutions, and an EcoRV site into pUC57. Substitutions in these inserts introduced AUG triplets at codons 252, 273, 291 and 304 of the 306 amino acid (a.a.)-long (35.2 kDa) RTCV coding sequence, to increase radiolabelling of the translation product. pUC57-T7-Stem-[RTCV nt. 210–1488]-MAC-STOP and pUC57-T7-Stem-[RTCV nt. 210–1488]-AUU-STOP were generated by substituting the fourth codon (UUU) in the ORF by a UAA stop codon, and AUG_534_ by a UGA stop codon, respectively.

The dicistronic transcription vectors pUC57[T7-(DC-Stem) RTCV nt1-1488(wt)] and pUC57[T7-(DC-Stem) RTCV nt1-1488(GGG_451-453_CCC)] were made by inserting DNA derived from pUC57-T7-Stem-[RTCV nt1-1488] and pUC57-T7-Stem-[RTCV nt1-1488](GGG_451–453_CCC) and flanked by 5′ XhoI and 3′ EcoRV sites into DC Aichivirus (23) to replace the Aichivirus IRES and ORF2 by a hairpin (5′-GGGCCCGACCCGGTGACGGGTCGGGCCC-3′) (Δ*G* = −32.40 kcal/mol) and RTCV nt. 1–1488, with or without GGG_451–453_CCC substitutions and modified as described above to introduce additional AUG triplets into ORF2.

pUC57 plasmids were linearized by EcoRV and pXL-HCV was linearized by EcoRI. mRNAs were transcribed with T7 RNA polymerase. [^32^P]-labelled RTCV nt.210–1488 mRNA was transcribed in the presence of [α-^32^P]GTP.

### Purification of initiation factors, ribosomal subunits and elongation factors

Ribosomal 40S and 60S subunits, native eIF2, eIF3, eIF5B, eEF1H, eEF2 and total aminoacyl-tRNA synthetases, were purified from RRL (Green Hectares, Oregon, WI), and recombinant eIF1, eIF1A, eIF4A^R362Q^ and eIF5 were expressed in *E. coli* BL21(DE3) and purified as described (52–60). Native total calf tRNA (Promega, Madison, WI) and *in vitro* transcribed tRNA_i_^Met^ were aminoacylated as described (58).

### Assembly and analysis of ribosomal complexes

Binary 40S/IRES and 48S complexes were assembled by incubating 0.5 pmol RTCV IRES mRNA with 3.3 pmol 40S subunits and combinations of 10 pmol eIF2, 4 pmol eIF3, 15 pmol eIF1, 15 pmol eIF1A and total native aa-tRNAs containing ∼2.5 Met-tRNA or 3.5 pmol *in vitro* transcribed Met-tRNA_i_^Met^ for 10 min at 37°C in 40 μL buffer A (20 mM Tris, pH 7.5, 100 mM KCl, 2 mM dithiothreitol, 0.25 mM spermidine, 2.5 mM MgCl_2_) supplemented with 1 mM ATP, 0.5 mM GTP and 30 U RNAse inhibitor.

To assay elongation, 48S complexes were supplemented with 4.3 pmol 60S subunits, 10 pmol eIF5 and 5 pmol eIF5B, and incubated for 10 min at 37°C. 3.75 pmol eIF1H and 4.5 pmol eEF2 were then added and incubation continued for an additional 10 min at 37°C.

Assembled 48S and 80S complexes were analysed by toe-printing using AMV reverse transcriptase and a γ^32^P-end-labelled primer (5′-TGAGGGTAGGAGGAGTAAAGC-3′) complementary to RTCV nt. 617–637.

### *In vitro* translation

Monocistronic or dicistronic mRNAs (4 pmol) were translated for 1 h at 37°C using the ‘optimized for translation and ready to use’ nuclease-treated rabbit reticulocyte lysate (Promega) in 20 μl reaction mixtures supplemented with 0.5 mCi/ml [^35^S]methionine (43.5 TBq/mmol) in the presence/absence of ∼20 pmol dominant-negative eIF4A^R362Q^, as indicated. Translation products were analysed by electrophoresis using Nu-PAGE 4–12% Bis–Tris-Gel (Invitrogen), followed by autoradiography.

### Analysis of ribosomal complexes by sucrose density gradient centrifugation

Ribosomal complexes assembled on ^32^P-labelled mRNA as described above in scaled-up 100 μl reaction mixtures were analysed by centrifugation through 10–30% sucrose density gradients prepared in buffer A in a Beckmann SW55 rotor at 53 000 rpm for 105 min. Ribosomal association of [^32^P]mRNA was measured by Cerenkov counting.

### Nucleotide sequence alignment and modelling of RNA structures

IRES sequences were aligned with Clustal Omega (http://www.ebi.ac.uk/Tools/msa/clustalo/) and adjusted manually using established IRES structures as guides (10,61,62). Calicivirus 5′UTR structures were modelled as described for picornavirus IRESs (11,13,63). Structural elements were modelled using Mfold (http://unafold.rna.albany.edu/?q=mfold) (64), pKiss (http://bibiserv2.cebitec.uni-bielefeld.de/pkiss) (65) and CentroidFold (http://www.ncrna.org/centroidfold) (66), using default parameters.

### Analysis of protein sequences

Sequences were aligned using MUSCLE (v3.8.31) with default settings. Gaps and ambiguously aligned regions were stripped using GBlock (v0.91b) (67) with default settings. Phylogenetic trees with 500 bootstrap resamples of the alignment data sets were generated using the maximum-likelihood method in PhyML3.1 (68). Bootstrap values for each node are given with a threshold of 70%. ORFs in the genome were predicted using ExPASy translate (https://web.expasy.org/translate/). Individual proteins were identified by comparison with reference FCV, MNV and RHDV ORF1 polyproteins.

## RESULTS

### Avian caliciviruses with long 5′UTRs form part of a discrete phylogenetic branch

**Figure 1.**
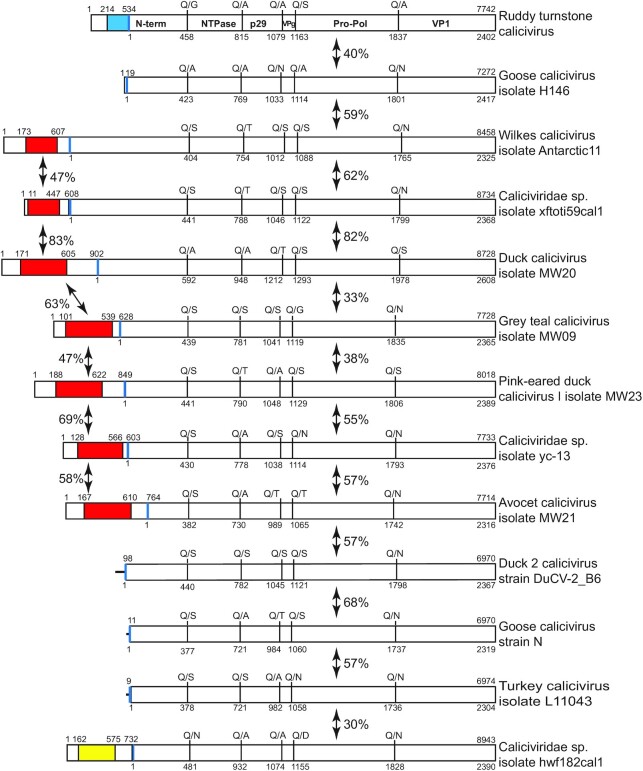
Schematic representation of the 5′UTR and ORF1 of the avian caliciviruses. Schematic representation of the 5′UTR and ORF1 of the avian caliciviruses ruddy turnstone calicivirus, goose calicivirus isolate H146, Wilkes calicivirus isolate Antarctic11, *Caliciviridae* sp. isolate xftoti59cal1, duck calicivirus isolate MW20, grey teal calicivirus isolate MW09, Pink-eared duck calicivirus I isolate MW23, *Caliciviridae* sp. isolate yc-13, avocet calicivirus isolate MW21, duck calicivirus 2 strain DuCV-2_B6, goose calicivirus strain N, turkey calicivirus isolate L11043 and *Caliciviridae* sp. isolate hwf182cal1. ORF1 is cleaved into mature structural and non-structural proteins (labelled in the RTCV sequence). The sequences of protease cleavage sites are indicated above each ORF, with amino acid numbering below. Extended 5′UTRs that contain a putative IRES are colored blue (type 4 IRES), red (type 2 IRES) and yellow (type 5 IRES). Sequence identity between polyproteins and between IRESs is indicated. The shaded areas in the type 2 IRESs correspond to domains H, I, J and K, and in the type 5 IRES to domains I, J, K and L.

**Figure 2.**
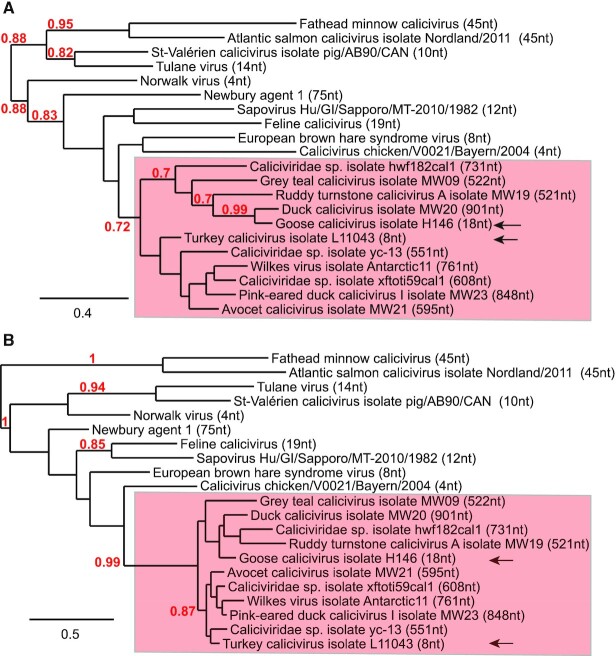
Phylogenetic analysis of members of *Caliciviridae* genera. Phylogenetic analysis based on the amino acid sequence of VP1 (**A**) and Pro-Pol (**B**) proteins in members of *Caliciviridae* genera. The viruses listed against a red background contain a putative IRES, except for the representative members of the genera *Nacovirus* and ‘*Sanovirus*’ that do not contain an extended 5′UTR, which are indicated by black arrows. The numbers at the branch nodes represent the bootstrap confidence levels (above 70%). Bar, (A) 0.3 and (B) 0.5 amino acid substitutions per site. The following viral sequences were analysed (name of each virus is followed by the corresponding Genbank accession number): fathead minnow calicivirus (genus *Minovirus*) (KX371097.1), Atlantic salmon calicivirus isolate Nordland/2011 (genus *Salovirus*) (KJ577139.1), St-Valérien calicivirus isolate pig/AB90/CAN (genus *Valovirus*) (FJ355928.1), Tulane virus (genus *Recovirus*) (EU391643.1), Norwalk virus (genus *Norovirus*) (M87661.2), Newbury agent 1 (genus *Nebovirus*) (DQ013304.1), sapovirus Hu/Dresden/pJG-Sap01/DE (genus *Sapovirus*) (HM002617.1), feline calicivirus (FCV) (genus *Vesivirus*) (M86379.1), European brown hare syndrome virus (genus *Lagovirus*) (Z69620.1), calicivirus chicken/V0021/Bayern/2004 (genus *Bavovirus*) (HQ010042.1), *Caliciviridae* sp. isolate hwf182cal1 (MT138020.1), grey teal calicivirus isolate MW09 (MK204392.1), ruddy turnstone calicivirus A isolate MW19 (MH453861.1), duck calicivirus isolate MW20 (MH453811.1), goose calicivirus isolate H146 (proposed genus ‘*Sanovirus*’) (KY399947.1), turkey calicivirus isolate L11043 (JQ347522.1), *Caliciviridae* sp. isolate yc-13 (KY312552.1), Wilkes virus isolate Antarctic11 (MT025075.1), *Caliciviridae* sp. isolate xftoti59cal1 (MT138028.1), pink-eared duck calicivirus I isolate MW23 (MK204416.1), and avocet calicivirus isolate MW21 (MH453804.1).

Several avian calicivirus genomes have exceptionally long 5′UTRs, including Wilkes virus, Avocet calicivirus (AvCV), duck calicivirus (DuCV) isolate MW20, grey teal calicivirus (GTCV), Pink-eared duck calicivirus 1 (PeDuCV1), *Caliciviridae* sp. isolate yc-13, ruddy turnstone calicivirus A (RTCV), *Caliciviridae* sp. isolate xftoti59cal1 and *Caliciviridae* sp. isolate hwf182cal1 (48–51). Caliciviruses characteristically have very short 5′UTRs (44), so that the relationship between the genomes of these viruses and of members of different calicivirus genera was first characterized. The avian ORF1 polyprotein sequences are closely related to each other (32%-85% sequence identity), although the length and sequence of the N-terminal protein differs significantly between genomes. Predicted protease cleavage sites in ORF1 of these viruses (Figure 1) corresponded closely to those in reference ORF1 polyproteins, including feline calicivirus (FCV) (69), murine norovirus (MNV) (70) and rabbit hemorrhagic disease virus (71). The VP1 sequence is the accepted standard for phylogenetic analysis of calicivirus genomes, and 3C protease/3D polymerase sequences are also informative and thus commonly used in such analysis (48–51). Here, phylogenetic analysis of VP1 (Figure 2A) and of the protease-polymerase precursor (Figure 2B) indicated that the genomes with long 5′UTRs are restricted to a single well-supported branch of *Caliciviridae*, together with members of the *Nacovirus* and ‘*Sanovirus*’ genera of *Caliciviridae* that have conventionally short 5′UTRs.

### Avian calivirus genomes contain candidate type 2, type 4 and type 5 IRESs

**Figure 3.**
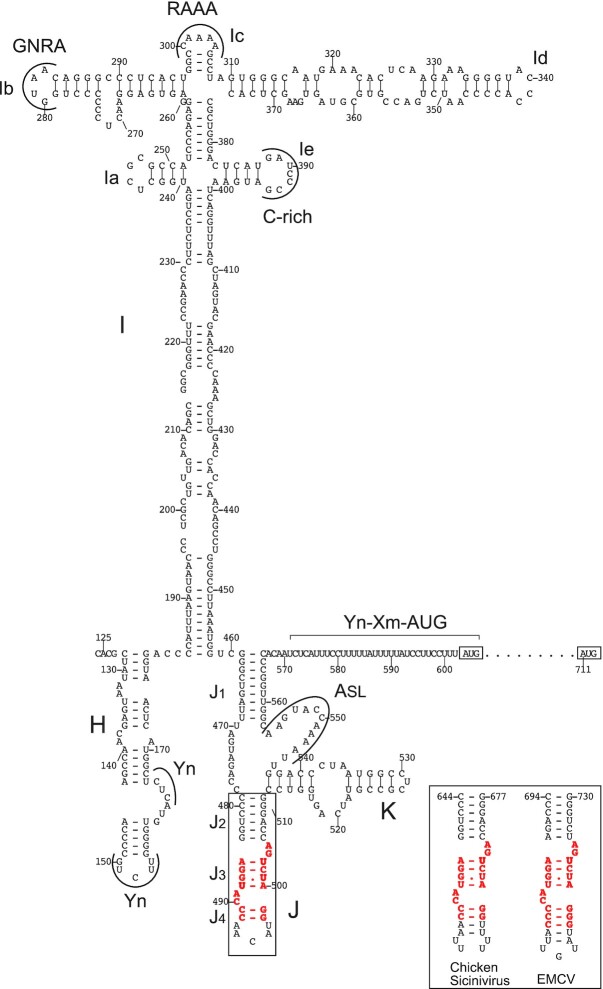
Model of the secondary structure of the *Caliciviridae* sp. isolate yc-13 IRES. Domains/subdomains in the core IRES are labelled sequentially from H to K; subdomains in domain I are labelled sequentially from Ia to Ie, and helices in domain J are labelled J_1_ to J_4_. The model is annotated to show conserved sequence motifs, including oligopyrimidine (Yn) motifs in domain H, GNRA, RAAA and C-rich motifs at the apex of domain I, the ASL domain in the J-K domain and the Yn-Xm-AUG motif downstream of the J–K domain. The nucleotides that make up the conserved discontinuous sequence motif present at the apex of domain J (11) are indicated in bold red font: equivalent motifs in domain J of the type 2 chicken Sicinivirus and EMCV IRESs are shown in the inset box. Nucleotides are numbered at 10-nucleotide intervals, and the initiation codons AUG_603_ and AUG_711_ are boxed.

The 5′UTRs of AvCV, DuCV, GTCV, PeDuCV, Wilkes virus and *Caliciviridae* sp. isolates yc-13 and Xftoti are related to each other and to the type 2 IRESs of the *Avisivirus*, *Gallivirus* and *Sicinivirus* genera of *Picornaviridae*. Although these caliciviruses are formally unclassified, our phylogenetic analysis (Figure 2) is consistent with suggestions (48,51) that they may be assigned to two genera: AvCV, PeDuCV, Wilkes virus and *Caliciviridae* sp. isolates yc-13 and Xftoti to the genus *Nacovirus* and DuCV and GTCV to the genus ‘*Sanovirus*’. The regions of greatest homology (65–67% nucleotide identity) correspond to most of the I domain and the entire J and K domains of Avisivirus A1 (72), Sicinivirus sp. strain RS/BR/2015/5R and chicken gallivirus 1 isolate 518C (62) IRESs. Pairwise sequence similarity between these putative calicivirus IRESs ranged from 43–68%, and ∼32% of nucleotides are fully conserved in all seven calicivirus 5′UTRs (Supplementary Figure S1). Consistently, they form type 2 IRES-like structures (e.g. Figure 3) with sequence motifs at locations that are known to be important for type 2 IRES function. These motifs include a pyrimidine-rich loop in domain H that interacts with PTB (73), a C-rich loop, a GNRA tetraloop and an AAA motif in apical arms of domain I (e.g. 74), an A-rich stem-loop that wedges between the minor grooves of the J and K domains (22), a bipartite sequence/ structural motif at the apex of domain J (11) and a 3′-terminal Yn-Xm-AUG motif (75).

**Figure 4.**
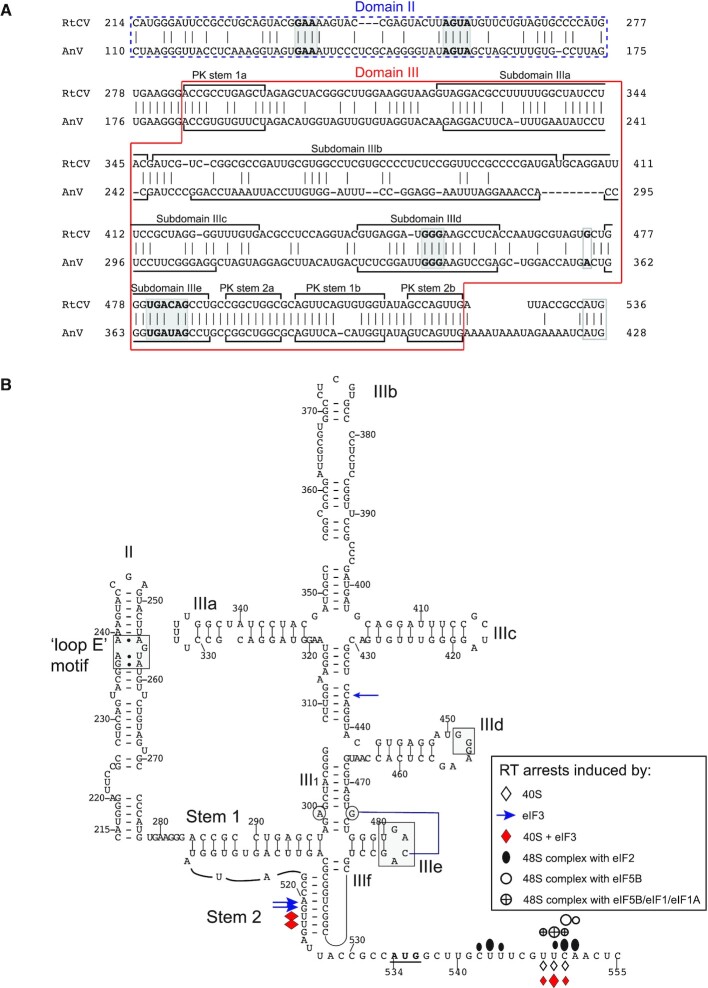
Model of the secondary structure of the ruddy turnstone calicivirus IRES. (**A**) sequence alignment of AV and RTCV IRESs, annotated to show the boundaries of predicted structural elements. Conserved loops are indicated by shading. (**B**) Model of the structure of the RTCV IRES, derived as described in the text. The AUG_534_ initiation codon for the ORF polyprotein is in bold and is indicated by a solid bar. The ‘loop E’ motif in domain II, conserved sequence motifs in the apical loops of domains IIId and IIIe and purine residues in helix III_1_ are indicated by gray shading. The tertiary base-pairing interaction between this purine residue and loop IIIe is indicated by a blue line. Sites at which primer extension was arrested by binding of eIF3, 40S ribosomal subunits and different ribosomal complexes are indicated by symbols (shown at the lower *right*). The size of symbols is proportional to the intensity of primer arrest.

The 525nt-long RTCV 5′UTR is unrelated to other calicivirus 5′UTRs, although phylogenetic analysis of VP1 and protease-polymerase precursor amino acid sequences suggests that RTCV might be assigned to the genus ‘*Sanovirus*’. However, nt. 170–525 are 60% identical to nt. 62–409 of the 5′UTR of Anativirus A (AnV) (Figure 4A) of the genus *Anativirus* (76) which form a type 4 IRES (10). The RTCV 5′UTR also shares strong sequence identity with type 4 IRESs from avian *Sapelovirus*-like picornaviruses (77–79), particularly with subdomains IIId, IIIe and the pseudoknot, the most strongly conserved elements of these IRESs (13). Consistently, modelling indicated that the structure of this region of the RTCV 5′UTR (Figure 4B) is closely related to the AnV type 4 IRES (10). Sequence differences between them are often covariant, so that the folding of structural elements is maintained by compensatory second site substitutions (Supplementary Figure S2). Both elements contain an HCV-like domain II with an internal loop near its base and GAA and AGUA sequences that form a ‘loop E’ motif. The apical IIIa, IIIb and IIIc subdomains in domain III form a four-way junction, although subdomain IIIa contains an ‘UUUUU’ loop instead of the apical ‘AGUA’ loop found in the HCV IRES. Domain IIId contains the apical GGG motif that engages with the ES7 element of 18S rRNA (29,35) and that is an invariant feature of all type 4 IRESs, and domain IIIe has an apical GACA tetraloop that could engage in a tertiary interaction with G_474_ (cf. 30) (Figure 4B). The pseudoknot at the base of RTCV domain III closely resembles the pseudoknot in the AnV IRES.

The 5′UTR of *Caliciviridae* sp. isolate hwf182cal1 (731 nt. long) shares a high level of nucleotide identity with elements of the 5′UTRs of Oscivirus A2 (80) and Passerivirus (27) that form type 5 IRESs. Homology extends from nt.162 at the 5′ border of domain I to the initiation codon, and reaches ∼60% nucleotide identity from nt. 215–667, which include domain J (equivalent to domain IV of type 1 IRESs), domain K (equivalent to domain J of type 2 IRESs), the polypyrimidine tract and domain L (11).

### The mechanism of initiation on the RTCV mRNA

**Figure 5.**
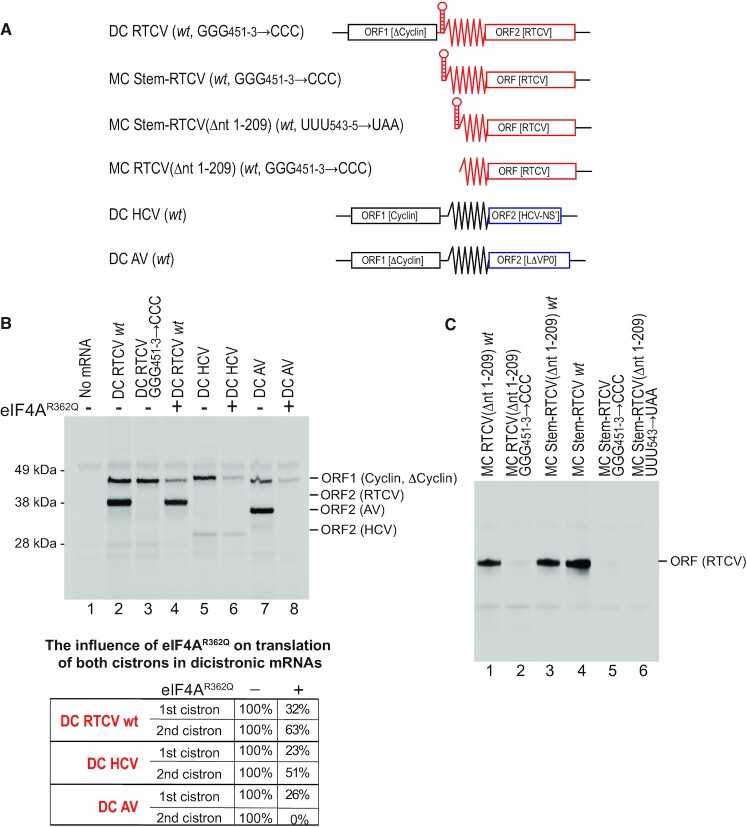
IRES function of the RTCV 5′UTR assayed by in vitro translation. (**A**) Schematic representations of monocistronic (MC) and dicistronic (DC) mRNA constructs with and without 5′-terminal stems and containing cyclin B2 (ORF1) and RTCV, HCV and AV 5′UTRs and adjacent coding regions (ORF2). (B, C) Representative gels showing translation in RRL of MC and DC *wt* and mutated RTCV mRNAs, as indicated, and of control DC HCV and DC AV mRNAs, (**B**) with and without inclusion of eIF4A^R362Q^. (B, lower panel) The influence of eIF4A^R362Q^ on translation of both cistrons in dicistronic mRNAs was quantified by Phosphorimager, with translation in the absence of eIF4A^R362Q^ defined as 100%.

We selected the RTCV 5′UTR, which contains a putative type 4 IRES, as a candidate to validate the identification and classification of putative IRESs in calicivirus 5′UTRs. To confirm that the RTCV 5′UTR contains an IRES, it was inserted between ORF1 and ORF2 in dicistronic DC RTCV mRNA (Figure 5A). To avoid any possibility of reinitiation after translation of the first cistron, the RTCV 5′UTR was also preceded by a stable 5′-terminal hairpin (Δ*G* = –32.4 kcal/mol) that prevents canonical initiation (81). The RTCV 5′UTR promoted efficient translation of the second cistron in rabbit reticulocyte lysate (RRL) (Figure 5B, lane 2), indicating that the RTCV 5′UTR is an IRES. To estimate the efficiency of translation mediated by the RTCV IRES, we compared it with the efficiency of type 4 HCV and type 5 AV IRESs in similar dicistronic mRNAs (Figure 5B, lanes 5 and 7). IRES activity was compared by assaying incorporation of ^35^S-Met during *in vitro* translation into ORF2 products, taking into consideration the methionine content of ORF2 in dicistronic RTCV, HCV and AV mRNAs, and assuming that the initiating methionine of translated proteins is cleaved off. Initiation by the RTCV IRES (defined as 100%) was similar to the AV IRES (∼90%) and substantially stronger that the HCV IRES (∼15%), consistent with reports that the HCV IRES is weaker than other type 4 IRESs (e.g. from classical swine fever virus (32)).

A defining characteristic of type 4 IRESs is that an essential G-rich loop in subdomain IIId engages with ES7 of 18S rRNA to promote and stabilize factor-independent binding of the IRES to the 40S subunit (e.g. 28,29,82,83). The strong loss of IRES activity resulting from GGG_451-3_→CCC substitutions in this motif (Figure 5B, lanes 2 and 3, and Figure 5C, compare lanes 1 and 4 with lanes 2 and 5) support the classification of the RTCV IRES as type 4. A second characteristic of type 4 IRESs is that, unlike type 1, type 2 and type 5 IRESs, their activity is independent of eIF4A and eIF4F (e.g. 32,34,82). We therefore compared the influence of the negative trans-dominant eIF4A^R362Q^ mutant on translation of the RTCV IRES with its influence on translation of type 4 HCV and type 5 AV IRESs in similar dicistronic mRNAs (Figure 5A). Like the HCV IRES (Figure 5B, lanes 5–6), translation promoted by the RTCV IRES (Figure 5B, lanes 2 and 4) was strongly resistant to inhibition by eIF4A^R362Q^, whereas initiation on the type 5 AV IRES was abrogated (Figure 5B, lanes 7–8) and initiation on the first cistron was strongly inhibited in all cases. Taken together, these functional characteristics of the RTCV IRES are consistent with its structure-based classification as a type 4 IRES.

When inserted into the monocistronic construct downstream of the same stable hairpin (Δ*G* = –32.4 kcal/mol) (MC-Stem-RTCV; Figure 5A), the RTCV IRES promoted translation in RRL as efficiently as in dicistronic DC RTCV mRNA (Figure 5C, lane 4). Deletion of nt. 1–209 of the 5′UTR in monocistronic constructs with or without the 5′-terminal stem, leaving only those sequences that are homologous to type 4 IRESs (MC-Stem-RTCV(Δnt 1–209) and MC-RTCV(Δnt 1–209); Figure 5A), did not impair IRES activity (Figure 5C, lanes 1 and 3). As in the case of the full-length RTCV IRES (Figure 5B, lane 3 and Figure 5C, lane 5), the activity of MC-RTCV(Δnt 1–209) mRNA was strongly impaired by the GGG_451-3_→CCC substitutions (Figure 5C, lane 2). Introduction of a stop codon after the first three sense codons of ORF2 in MC-RTCV(Δnt 1–209) (UUU_543–545_→UAA) mRNA abrogated synthesis of the 35.2 kDa ORF2 translation product (Figure 5C, lane 6), confirming that initiation on this mRNA occurred from the correct start site. These observations indicate that nt. 1–209 are not essential for the activity of the RTCV IRES.

Further characterization of the mechanism of initiation on the RTCV IRES was done by *in vitro* reconstitution. In this approach, ribosomal complexes are assembled from individual translational components (mRNA, ribosomal subunits, translation factors and aa-tRNAs), after which the ribosomal position on mRNA is determined by toe-printing. Ribosomal 48S/80S complexes with an established codon-anticodon interaction yield characteristic toe-prints ∼15–17 nt downstream of the P-site codon.

**Figure 6.**
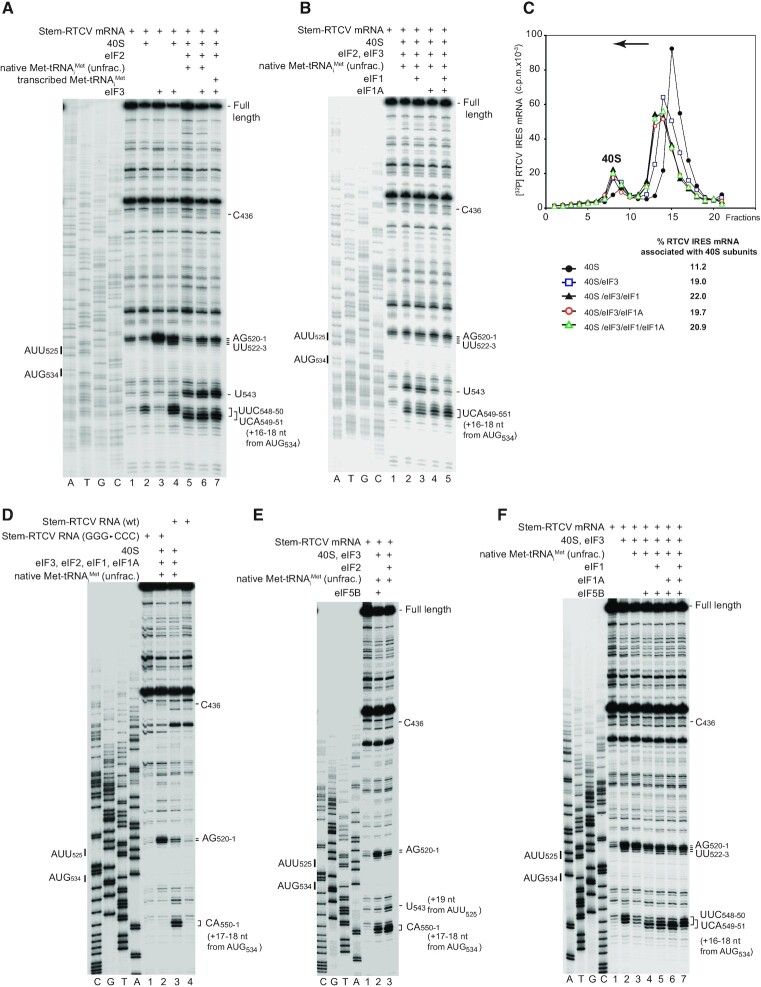
The mechanism of 48S complex formation on the RTCV IRES. (**A**, **B**, **D**–**F**) Toeprinting analysis of initiation complex formation on (A, B, D–F) *wt* and (D) GGG_451–453_→CCC mutant RTCV IRESs from translation components as indicated. The position of the RTCV initiation codon AUG_534_ and a near-cognate codon AUU_525_ are indicated on the left. Toeprints induced by binding of initiation factors and ribosomal complexes are indicated on the right. Toeprints at C_436_ and A_520_ are characteristic of bound eIF3. The positions of toeprints attributed to ribosomal complexes are shown relative to the mRNA codon in the ribosomal P-site. (**C**) Representative sucrose density gradient assays of binding of 40S subunits to the [^32^P]GTP-labelled RTCV IRES in the presence of initiation factors as indicated. Ribosomal complexes were fractionated by centrifugation in 10–30% linear sucrose density gradient and analysed by Cerenkov counting. The arrow indicates that sedimentation was from right to left. The position of 40S subunits is indicated. The percentage of the RTCV IRES mRNA bound to 40S subunits is shown.

The RTCV IRES bound directly to 40S subunits, yielding stable complexes that induced RT stops at nt. 548–550 (^+^15–17 relative to A (^+^1) of the initiation codon AUG_534_; Figure 6A, lane 2). This result indicates that like in other Type 4 IRESs, the coding region of the RTCV mRNA is correctly fixed in the mRNA-binding cleft of the 40S subunit to position AUG_534_ for base-pairing with initiator tRNA in the ribosomal P site. The RTCV IRES also bound directly to eIF3, leading to a reverse transcriptase (RT) stop at C_436_ between the IIIc and IIId subdomains (Figure 6A, lane 3; Figure 4B). The toe-print at this position is analogous to those induced by binding of eIF3 to HCV and other type 4 IRESs (32–34,82). Strikingly, binding of eIF3 to the RTCV IRES also strongly enhanced endogenous stops at AG_520-1_ in PK stem 2 (Figure 6A, lane 3; Figure 4B), indicating that eIF3 globally stabilizes the structure of this IRES. eIF3 also strongly enhanced formation of IRES/40S complexes. Thus, inclusion of eIF3 with 40S subunits intensified toe-prints +16–17 nt downstream from AUG_534_ and led to the appearance of toe-prints at nt. 522–523 in addition to those at nt. 520–521 induced by eIF3 alone (Figure 6A, lane 4; Figure 4B). The nt. 522–523 toe-prints map to PK stem 2 (Figure 4B), which interacts with ribosomal protein rpS28 in ribosomal complexes assembled on the CFSV and HCV IRESs (35,84). The appearance of these toe-prints would be consistent with eIF3-induced stabilization of the interaction of 40S subunits with the PK.

Inclusion of eIF2 and Met-tRNA_i_^Met^ in reaction mixtures containing 40S subunits (with or without eIF3) led to a small shift forward of toe-prints from UUC_548-550_ (^+^15-^+^17) to UCA_549–551_ (^+^16-^+^18) (Figure 6A, lanes 5–7), which is characteristic of 48S complex formation on type 4 IRESs and reflects localized adjustments of mRNA and the Met-tRNA_i_^Met^ anticodon in the mRNA-binding cleft (e.g. 32,34). It also yielded an additional strong toe-print at U_543_, which most likely resulted from 48S complex formation at the upstream near-cognate AUU_525_ codon (Figure 6A, lanes 5–7). 48S complex formation was more efficient in the presence of eIF3 (Figure 6A, compare lane 5 with lanes 6–7) and, unlike on some other IRESs (85), was not sensitive to replacement of native initiator tRNA by its *in vitro* transcribed version (Figure 6A, compare lanes 6 and 7). To our surprise, the relative efficiency of 48S complex formation on cognate AUG_534_ and upstream near-cognate AUU_525_ was not affected by inclusion of eIF1 alone (Figure 6B, compare lanes 2 and 3), whereas eIF1A alone or with eIF1 weakened the toe-print at AUU_525_ and enhanced 48S complex formation on AUG_534_ (Figure 6B, compare lane 2 with lanes 4, 5). In sucrose density gradient centrifugation experiments, eIF3 stimulated association of the IRES with 40S subunits ∼2-fold, but we did not observe further enhancement of 40S/IRES complex formation by inclusion of eIF1 and eIF1A over that promoted by eIF3 alone, implying that the role of eIF1 and eIF1A in initiation on this IRES is likely limited to ensuring the fidelity of initiation codon selection (Figure 6C).

Binding of type 4 IRESs to the 40S subunit depends on the initial establishment of base-pairing between the G-rich apical loop of subdomain IIId and ES7 of 18S rRNA (28,29,82,83). The substitution of the apical GGG motif in the IIId loop of the RTCV IRES by a CCC triplet abolished its ability to promote translation *in vitro* (Figure 5C) and, as expected, abrogated the appearance of toe-prints that are characteristic of 40S subunit association and 48S complex formation on the IRES (Figure 6D, lane 2). The apical loop of subdomain IIId is thus a critical determinant of ribosomal recruitment to this IRES. In contrast, this apical GGG loop does not influence binding of eIF3 to CSFV and other type 4 IRESs (28), and consistently, its substitution by a CCC triplet did not affect the eIF3-induced toe-prints (Figure 6D).

Viral infection activates innate immune responses, including phosphorylation of eIF2 and consequent impairment of translation (16). However, Type 4 CSFV and HCV IRESs retain initiation activity in these circumstances, at least in part due to their ability to utilize an alternative initiation mechanism in which eIF5B promotes binding of Met-tRNA_i_^Met^ to the IRES/40S subunit complex (37,38,86). We therefore investigated whether eIF5B can replace eIF2 in initiation on the RTCV IRES. In reaction mixtures containing 40S subunits, eIF3 and Met-tRNA_i_^Met^, eIF5B was able to promote 48S complex formation on AUG_534_, albeit at a lower level than with eIF2 (Figure 6E). In contrast to eIF2, eIF5B did not promote strengthening of toe-prints at U_543_ that likely corresponds to 48S complex formation on the upstream near-cognate codon AUU_525_ (Figure 6E). Inclusion into reaction mixtures of individual eIF1 and particularly of eIF1A slightly stimulated 48S complex formation on AUG_534_, whereas together, they substantially weakened toe-prints corresponding to 48S complexes formed on AUG_534_ and induced strong ^+^15-^+^17 nt toe-prints that are indicative of the formation of binary 40S/IRES complexes (Figure 6F). Thus, similarly to CSFV and SPV9 IRESs (37,82), eIF5B-mediated initiation on the RTCV IRES was sensitive to inhibition by eIF1 and eIF1A.

**Figure 7.**
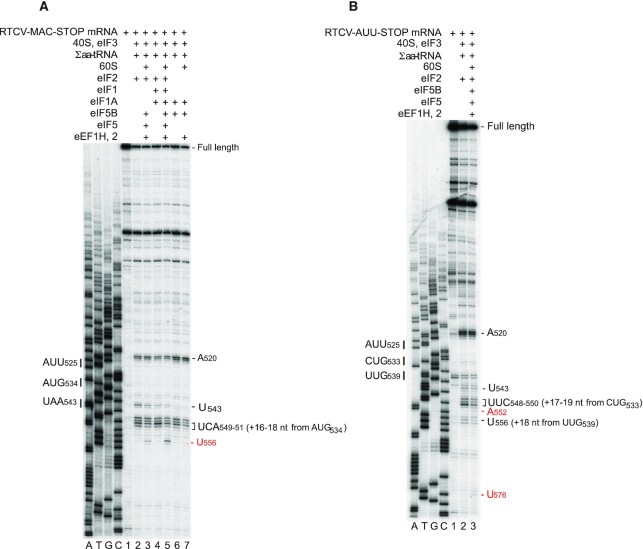
Elongation competence of 80S ribosomes assembled on the RTCV IRES. Toeprinting analysis of 48S initiation complexes, 80S initiation complexes and 80S ribosomal elongation complexes assembled on (**A**) RTCV-MAC-STOP and (**B**) RTCV-AUU STOP mRNAs from translation components as indicated. Toeprints corresponding to assembled ribosomal complexes are indicated on the right. Toeprints at A_520_ are characteristic of bound eIF3. The initiation codon AUG_534_ and the stop codon UAA_543_ are indicated on the left in panel (A) and the near-cognate initiation codons AUU_525_, CUG_533_ and UUG_539_ are indicated on the left in panel (B). Lanes A, T, C and G show the cDNA sequence corresponding to RTCV mRNA.

To confirm that 40S ribosomal complexes formed on the RTCV IRES in the presence of Met-tRNA_i_^Met^ and either eIF2 or eIF5B are *bona fide* 48S initiation complexes, we tested their ability to join 60S subunits to form elongation-competent 80S ribosomes. For this, we employed RTCV-MAC-STOP mRNA, in which a UAA stop codon was introduced as the third codon downstream of AUG_534_. Addition of 60S subunits, eIF5, eIF5B, elongation factor (eEF) 1H, eEF2, and total aminoacylated tRNAs (Σaa-tRNA) to 48S complexes that had been assembled with eIF2 and eIF3 (with/without eIF1 and eIF1A) led to the appearance of a toe-print at U_556_, six nucleotides downstream of the toe-prints corresponding to 48S complexes at AUG_534_, and to a concomitant decrease in the intensity of 48S toe-prints (Figure 7A, lanes 3 and 5). The appearance of the U_556_ toe-print is consistent with the expected occurrence of two programmed elongation events leading to formation of pre-termination complexes (pre-TCs). The higher efficiency of 48S complex formation in the presence of eIF1 and eIF1A correlated with the higher intensity of toe-prints corresponding to pre-TCs assembled in their presence. 40S ribosomal complexes assembled with Met-tRNA_i_^Met^, eIF5B, eIF3 and eIF1A also underwent elongation and formation of pre-TCs upon addition of 60S subunits, elongation factors and Σaa-tRNA, albeit with substantially lower efficiency (Figure 7A, lane 7).

Next, we assayed the elongation competency of 40S ribosomal complexes that presumably formed on the near-cognate AUU_525_ codon (particularly in the absence of eIF1 and eIF1A) and were characterized by the strong U_543_ toe-print (Figure 6A, B). For this, AUG_534_, which is the fourth in-frame codon after AUU_525_, was replaced by a UGA stop codon to allow synthesis of a tripeptide after initiation on AUU_525_. However, analysis of 48S complexes assembled on this mRNA in reaction mixtures containing 40S subunits, Met-tRNA_i_^Met^, eIF2 and eIF3 revealed not only the U_543_ toe-print ^+^18nt downstream of AUU_525_, but also strong stops at UUC_548-550_ and a weaker stop at U_556_, which are suggestive of 48S complex formation at the near-cognate codons CUG_533_ and UUG_539_, respectively (Figure 7B, lane 2). Addition of 60S subunits, eIF5, eIF5B, eEF1H, eEF2 and Σaa-tRNA did not substantially reduce the intensity of toe-prints corresponding to 40S complexes formed on AUU_525_ and the concomitant appearance/ strengthening of toe-prints indicating assembly of post-TCs on UGA_534_ (Figure 7B, lane 3). However, it led to weakening of the toe-prints at UUC_548-550_ and U_556_ corresponding to 48S complexes formed on CUG_533_ and UUG_539_, and to the appearance of toe-prints at A_552_ and U_576_ (Figure 7B, lane 3), which would be consistent with both complexes having begun elongation and then having arrested at specific codons due to shortage of corresponding aa-tRNAs in the unfractionated Σaa-tRNA mixture (87). We conclude that in the absence of eIF1 and eIF1A, elongation-competent initiation complexes can form on the RTCV IRES at near-cognate initiation codons in the immediate vicinity of the initiation codon AUG_534_.

Taken together, these data confirm that the RTCV 5′UTR contains a fully functional type 4 IRES.

## DISCUSSION

### Horizontal gene transfer (HGT) of IRES elements

We report that several avian calicivirus genomes contain 5′UTR elements that can confidently be assigned to established classes of viral IRES. They share a high level of sequence identity with IRESs from specific picornaviruses and may therefore have a common evolutionary origin. The calicivirus type 2 and type 5 IRESs constitute the first examples of these types of IRES in viral genomes outside the *Picornaviridae*, and the identification of the RTCV IRES is evidence of the co-option of type 4 IRESs by a third virus family in addition to *Flaviviridae* and *Picornaviridae*. These findings provide further evidence that IRESs can be exchanged between viral families by HGT (15). These observations were unexpected because FCV genomic RNAs that were engineered to contain a 5′-terminal EMCV IRES were not infectious, and equivalent hybrid MNV RNAs were infectious but yielded progeny in which the IRES had been lost and the 5′-end of the genome had been precisely regenerated (88). Avian calicivirus genomes therefore likely have properties that permit retention of type 2 IRESs, as discussed below. Recombination is an established feature of calicivirus evolution and occurs most commonly at the junction of ORF1 (nonstructural proteins) and ORF2 (structural proteins) (89–91). The present report suggests that an additional important breakpoint maps to the vicinity of the junction of the 5′UTR and ORF1.

The presence of type 4 IRESs in the genomes of members of *Caliciviridae*, *Flaviviridae* and *Picornaviridae* is indicative of their ability to function in different environments and genomic contexts, which likely reflects their strong activity, their modular, self-contained nature and their exploitation of highly conserved binding targets on components of the translation apparatus that occur in a wide range of organisms. For example, the UCCC loop of ES7 of 18S rRNA, which base-pairs with the GGG motif at the apex of IRES domain IIId, occurs in mammals, birds, reptiles, amphibians and fish (13,35). Interestingly, this element of 18S rRNA is also exploited by the termination-reinitiation process that leads to translation of the minor capsid protein from calicivirus subgenomic mRNA (42,43).

HGT involving type 2 IRESs has been detected less frequently, but it has been implicated in the formation of the genomes of members of the *Rabovirus* genus of *Picornaviridae* (92,93). The identification of type 2 IRESs in seven distinct avian caliciviruses suggests that these elements can readily be exchanged between unrelated viral genomes. HGT of type 2 and type 4 IRESs likely have the same requirements, namely the ability of the IRES to function as a self-contained unit and the presence of conserved IRES-binding surfaces on components of the translation apparatus in different species. Initiation on mammalian type 2 IRESs depends on their interaction with eIF4G (19–22), and type 2 IRESs from avian viruses likely interact analogously with avian eIF4G. The central IRES-binding domain of human eIF4G is closely related to the equivalent domain in eIF4G from the *Gruidiae* (i.e. cranes) and *Anatidae* (ducks, geese and swans) (∼75–95% amino acid identity, including all basic and aromatic residues that have been implicated in this interaction (21,22)).

The presence of type 2 IRESs in numerous avian calicivirus genomes contrasts with the incompatibility of the (type 2) EMCV IRES with FCV and MNV genomes, and its precise elimination from chimeric EMCV-MNV genomes (88). An appealling hypothesis is that this incompatibility is due to deleterious interference between the translation initiation processes mediated by the EMCV IRES and by FCV and MNV VPgs, respectively. These processes both depend on specific interactions with eIF4F: type 2 IRESs bind eIF4G/eIF4A, the MNV VPg promotes initiation via interaction with eIF4G, whereas the FCV VPg interacts with eIF4E (45–47). A notable feature of avian IRES-containing caliciviruses is that they encode a VPg protein (74–84 a.a. long) that is considerably shorter than FCV and MNV VPgs (111 a.a. and 124 a.a., respectively). Whereas the eIF4E-binding determinants in the former have not been established, interaction of eIF4G with the latter involves C-terminal elements (45,46) that are absent from the VPgs of IRES-containing and related caliciviruses (Supplementary Figure S3). The ‘short’ calicivirus VPgs may thus not compete with type 2 and type 5 IRESs for binding to eIF4G. It is currently not possible to distinguish between scenarios in which the presence of a ‘short’ VPg either provides an environment that is permissive for IRES acquisition by HGT or reflects evolutionary loss of initiation factor-binding determinants that are not needed for or even interfere with IRES function.

### Consequences of horizontal gene transfer of IRESs into calicivirus genomes

Type 2, type 4 and type 5 IRESs have well-characterized functions, and their acquisition by HGT would likely alter the gene expression strategy of recombinant calicivirus progeny relative to the parental strain. Calicivirus infection leads to a shut-off of cellular translation and to multiple changes to the cellular translation apparatus, including phosphorylation of eIF2α, viral protease-mediated cleavage of the poly(A)-binding protein PABP, eIF4E phosphorylation, induced caspase-mediated cleavage of eIF4G and translocation of PTB from the nucleus to the cytoplasm (47,94–96). Adoption by a calicivirus of an alternative mechanism for translation initiation, such as that mediated by type 2 IRESs, which is independent of eIF4E (19) or by type 4 IRESs, which is independent of eIF4F (32,34) might therefore alter viral replication kinetics and virus yield. Calicivirus replication kinetics might also be affected by the acquisition of a type 2 or a type 5 IRES because whereas their function is commonly PTB-dependent (11,18,20,23), translation of FCV mRNA and thus potentially of other caliciviruses is inhibited by PTB (93).

### The structure of the RTCV type 4 IRES

The RTCV genome is the first from a virus outside the *Flaviviridae* and *Picornaviridae* in which a type 4 IRES has been identified. It contains all but one of the characteristic structural elements and sequence motifs of conventional type 4 IRES, including a sub-apical ‘loop E’ motif in domain II, a pseudoknot at the base of domain III and a series of stem–loops, designated IIIa to IIIf. Subdomain IIId has a functionally important apical GGG motif that is critical for binding of the IRES to the 40S subunit (Figures 5B, 5C, 6D) and that, by analogy with type 4 CSFV and HCV IRESs, engages with ES7 of 18S rRNA (29,35). Subdomain IIIa lacks the ‘AGUA’ loop that occurs in the HCV IRES, and instead contains a ‘UUUUU’ loop like that in the type 4 IRESs in members of the avian *Colbovirus*, *Megrivirus* and *Mesivirus* genera of *Picornaviridae* (13). eIF3 binds to the junction region of domain III (33,35), but whether this U-rich loop constitutes an adaptation to the avian translation apparatus remains to be determined.

### The mechanism of initiation on the RTCV type 4 IRES

The resistance of RTCV IRES-mediated translation to inhibition by eIF4A^R362Q^ (Figure 5B) is characteristic of type 4 IRES (32,34,41,82) and is consistent with the mechanism of initiation that was elucidated here by *in vitro* reconstitution. The RTCV IRES bound directly and stably to 40S subunits and to eIF3, and could support recruitment of Met-tRNA_i_^Met^ either as part of the eIF2-TC (in which case subunit joining mediated by eIF5 and eIF5B yielded an elongation-competent 80S ribosome) or via eIF5B. The RTCV IRES can therefore initiate translation without the involvement of group 4 eIFs, using a mechanism that is typical of type 4 IRESs.

Nevertheless, some aspects of the initiation process characterized here are notable and may be generally relevant to this class of IRES. Although eIF3 binds to the apical region of domain III, yielding toeprints at C_436_ that are directly comparable to those that appear on binding of eIF3 to HCV, CSFV, BVDV and *Sapelovirus A* IRESs (32–34,82), it nevertheless influenced distant elements of the IRES, stabilizing PK Stem 2 and its interaction with the 40S subunit (Figure 6A). This stem directs the initiation codon and ORF into the mRNA binding cleft of the 40S subunit (31), and eIF3 therefore indirectly promotes this interaction. This activity may therefore contribute to eIF3′s stimulatory effect on initiation on type 4 IRESs.

On the *wt* RTCV IRES, 48S complexes could presumably form at AUU_525_, upstream of the initiation codon AUG_534_ (Figure 6A, B), and on the [AUG_534_UGA] mutant IRES, 48S complexes also assembled at CUG_533_ and UUG_539_ (Figure 7B). These observations suggest that binding of IRES sequences downstream of PK stem 2 in the mRNA channel of the 40S subunit is followed by a one-dimensional search for the initiation codon, i.e. scanning. We note that the length of the spacer between the PK and the initiation codon varies from 8 to 17 nt in different type 4 IRESs (10,13) and that initiation codon location and selection on them therefore likely involves limited localized scanning and is not simply enforced by a molecular ruler mechanism as a function of distance from the PK. Thus, final adjustments of the positioning of the spacer in this and other IRESs in the mRNA channel should occur upon establishment of base-pairing between the Met-tRNA_i_^Met^ anticodon and the initiation codon. Notably, appearance of toe-printing stops corresponding to 48S complex formation on the near-by upstream AUG was observed on a variant of the CSFV IRES lacking domain II (37). Thus, CSFV domain II functions to fix the spacer and initiation codon in the mRNA-binding channel, influencing initiation codon selection. Domain II of the RTCV IRES may be deficient in this function, potentially allowing initiation to occur in alternate sites around AUG_534_.

## DATA AVAILABILITY

All data are available in the main text or the supplementary materials.

## Supplementary Material

gkab1243_Supplemental_File
