# Supplementary material for: Horizontal gene transfer as a mechanism for the promiscuous acquisition of distinct classes of IRES by avian caliciviruses
Source: Nucleic Acids Res. 2021 Dec 20;50(2):1052–68. doi: 10.1093/nar/gkab1243 (PMC8789048; doi:10.1093/nar/gkab1243)
Supplement: gkab1243_Supplemental_File [file gkab1243_supplemental_file.pdf]

Domain H

```
                                       Yn                  Yn
Crane      128  CTATAATGAGCAACCGAACCCCGTCTTGGGG.TGTACTCTCGGTACTCAATGG  ACC   182
Avocet     167 TCTAACGTATCTCCCTCCCCACACTCTTTGTGGGATGCTCTGGG-AGACGATGG  ACC   222
Wilkes     173 TTTAATGATGTTG..GCACCGCAACCTT....GCGGCCTTGCTGGTATCAATGG  ACC   223
Pink-eared 188  CTAAGGTGGCTGGAACAGC...AACTT....GCTGTCTCTCTGGCTGCCATGG  ATC   236
Xftoti      11  TTAAGGCTGCTGG..CACCGCCGTCTT...GGCGGTCTCGCTGGCATCCATGG  ACC    61
Duck       171 CCTATACACCTGGCA.....CCACTTTT....GTGGTCTCGC--TGGGTGAAGG   CC   215
Grey Teal  101 GCTATACACATCCCCTGC....ACTCTTT.......GCTCTGGG-ATGTGAAGGC   C   144
```

Domain I

```
Crane      183  CCATTTAAG.TAACCCTCGCTGTTGAC.ACAGCGGCGGGTTTCCGAACCCTTCTCCTGA. 239
Avocet     223  CTAACTGACTAACCTTGGTACGACGACACTGTCGTCGCATCA.CGAACACACTTCTTGTG 264
Wilkes     224  CCTACCACCTAACCC.ACTCACTTGACACATGTGGTTCGCCCACGAAACCCCCT.TTGTG 281
Pink-eared 237  CATATTCTG.TAACCCTGTGCGTTGACGCGCATGGAGTTACTCCGAACCCCCTT.TTGCG 294
Xftoti      62  CTAACACTGTCATCC.TGCCTGTTGACACAGGTAGGGCTATGACGAACACCCTT.TTGTG 119
Duck       216 CCATTGGCCG.TCTGGTCACAGCACGGCCCAACCTAGTACCCA.CGAACGCACCCAA...C 271
Grey Teal  145 CCGTTGCTCTTGCAACGGCAAGTATGGCGTAGCCTAGTACTGG.CGAACGTCACTCATGCG 204

                                                  C-rich     GNRA
Crane      240 TG.GC.TCGCGCCA.TCCCAGAGGAGTGAGGAACTCCCCCTGGTAACAGGCCCTCACT 296
Avocet     265 TG.GC.TCGCGCCA.TCCAAGAGGAGTTGGAACTCCCCCACGGTAACGTGGCCCCAACT 179
Wilkes     282 TG.GC.TAGCGCCA.TCTGAGAGGAGTGCGGAACTCCCCCACGGTAACGTGGCCCGCACT 338
Pink-eared 295 TG.GC.TCGCGCCA.TCCAAGAGGAGTGTGGAACCCCCCCACGGTAACGTGGCCCACACT 351
Xftoti     120 TG.GC.TCGCGCCA.TCCAAGAGGAGTAGGGAACTCCCCCTGGTAACAGGCCCCTACT 176
Duck       272 TGAGCGTCGCGGCG.TC.ATGAGGCGGGCGGAGCACCCCCCTGGTAACAGGTCCTCCCG 329
Grey Teal  205 TG.TC.TCGCGACGCGTAGTGAGG.GTGTGGAAACCCCACGTGGTAACACGTGCCACACT 261

       AAA
Crane      297 GGCCAAAAGCCTAGTGGGCAATGAAACACTCAAGAAAGGGGTACCCACCCCAATCTGACC 356
Avocet     180 GGCCTAAAGCCTAGTGTGCAATCAACATGCCTAGTCGGCTGTACCCACGGCCTGGCTTAC 239
Wilkes     339 GGCCTAAAGCCTAGTGTGCAAGGACACATGCTGGTCGGTTGTACCCACGGCCTGCCTACC 398
Pink-eared 352 GGCCTAAAGCCTAGTGTGCAGGATTGCTGCCTGGACGGCTGTACCCACGGCCTGTCCTAC 411
Xftoti     177 GGCCAAAAGCCTAGTGTGCAGGAATTAAGTCTAGTCGGCTGTACCCACGGCCTGGCTTAC 236
Duck       330 GGCCACAAGCCACGTGGGCAGGGCGAAGGTGAAGGAAGGGTCCCCCGACCCAACCT.CAA 388
Grey Teal  262 GGCCAAAAGCCAAGTGGACAGGGTGAATACTGCAGGGAGCTGTACCCACGGCTCCTCTAA 321

                                                 C-rich
Crane      357 GT.GCGTAGTGAAGCTCACCGCTGGGA.CTCATGATCCCGATGA.ATCA.GGTTTA      408
Avocet     240 GTATGTTGGTGAAGCTCACCGCTTGGA..CTCCGACCCCGGAA...CACAAAAGT      296
Wilkes     399 AT.GTGTCCTGAAGCTCACCGCTCAGAC..CTCGATCCCGGAGA...CACACTGGG      448
Pink-ear   412 GTAGTTGTCCGAAGCTCACCGCTTGGA..CTCCGACCCCGGAA...CGCAACTGG      462
Xftoti     237 GCTTAAGACCGAAGCTCACCGCTTGGA.CTCCCGACCCCGGAA...CACAACTGG      287
Duck       389 GCCTGAACCCGC.GTTCACCCTCATGATCAGGCAAACCCAGCCTG.GCGGTTGTTT      442
Grey Teal  322 GTTGAAGCCCGA.GTTCACCCACTACGTTGTGCTA-CCCCGCACAGCGCCTGTTTG      375

Crane      409 GCTAGTACGAACCCCAAAGCTGG.ACCACCAACA..GCCTGGG.CCTTAAATGG   TC   460
Avocet     450 GCTAGTACTGCTGTGGCTTCAGG.ACCACCGTGTCCCAAGGCT.GACAGTTAG  CCCC   504
Wilkes     449 GCTAGTACTGTGCGCGCCGCCTGGACCACCAAGTCCTTGGGCA..GTGCGTAGG   AC   502
Pink-eared 463 GCTAGTACGACTAGCTCTTTGTGAACCACCAACCCACAGGA...ACAGCATATG   CT   515
Xftoti     288 GCTAGTAATCCTAGCTCTCCCTGGACCACCAACTTGCAGGACC...AGCGTTAG   CT   340
Duck       443 GTTAGTATTGGCGAAAAGCTGGC.ACCGACTGTTCCCAAGACCACCCAATGG    CAA   496
Grey Teal  376 ACTAGTATCCACGAAACGCTACC.CCCAGCTTGCCCGCGTGACTGACAACGG    CAC   429
```

Domain J-K

```
                                          Bipartite motif
Crane      461  GGGCTGATTTAGTAGACCCCCTGGAGGTACCC.AACATGGATCTGACCAGGGCCTGG    516
Avocet     505   CCCCGGCTTAGTAGACCCCCTGGAGGTACCCCTAACGGGATCTGACCAGGGCCACT    560
Wilkes     503  TGCCAGCCTTAGTAGACCCCCAGCAGGTACCC..CCTTGGATCTGAGCTGGGCCCTG    557
Pink-eared 516  GTCCTGTTCTAGTAGACCCCCTGGAGGTACCCTTCACAGGATCTGACCAGGGCCTGG    572
Xftoti     341  GGTCTGTCTTAGTAGACCCCCTGCAGGTACCCCGAAAGGGATCTGAGCAGGGCCTGG    397
Duck       497 GGGGGCTGGTGAGTAGATGCCCTGCAGGTACCC..TTTCGGATCTGAGCAGGGGCCCT    552
Grey Teal  430 TGGGTTTGGCGAGTAGATGCCCTGGAGGTACCC.ACTCTGGATCTGACCAGGGGCCCG    486

                                      ASL
Crane      517 TGACTATGCCGCTCCGGTAATCCCAGGTTAAAA.CCATGAACGGTTGGCCC         566
Avocet     561 TGACTATACCGATCCGGTAATGAGTGGTTAAAA.TAATGAACGGCTGGGGG         610
Wilkes     55  TGACTATACCGTTCCGGTAATGCAGGGTTAGAA.AAATGAACGGGTTGGCA         607
Pink-eared 573 TGACTATACCGCTCCGGTAATCCCAGGTTAAAA.CCATGAACGAATAGGAC         622
Xftoti     398 TGACTATACCGCTCCGGTAATCCCAGGTTAAAA.CCATGAACGGATAGGCC         447
Duck       553 TGACTATACCGTTCCGGTAAACAGGGGGTAAGAACCATGAAAGCGCAGCCCCC       605
Grey Teal  487 TGACTATACCGCTCCGGTAAACCGGGTATAAAAACCATGAAAGTGCAAACCCA       539
```

Supplementary Figure 1. Sequence alignment of the conserved core of calicivirus type 2 IRESs.

Sequence alignment of elements corresponding to IRES domains H, I and J-K in the 5'UTRs of *Caliciviridae* sp. isolate yc-13 ('Crane'), avocet calicivirus isolate MW21 ('Avocet'), Wilkes calicivirus isolate Antarctic11 ('Wilkes'), Pink-eared duck calicivirus I isolate MW23 ('Pink-eared'), *Caliciviridae* sp. isolate xftoti59cal1 ('xftoti'), duck calicivirus MW20 ('Duck') and grey teal calicivirus isolate MW09 ('Grey Teal'). Nucleotides that are identical in six or more of the seven sequences are indicated in bold, and motifs containing these conserved nucleotides are shaded blue. Sequences were annotated after alignment to show boundaries of predicted domains and sequence motifs that are characteristic of type 2 IRESs.

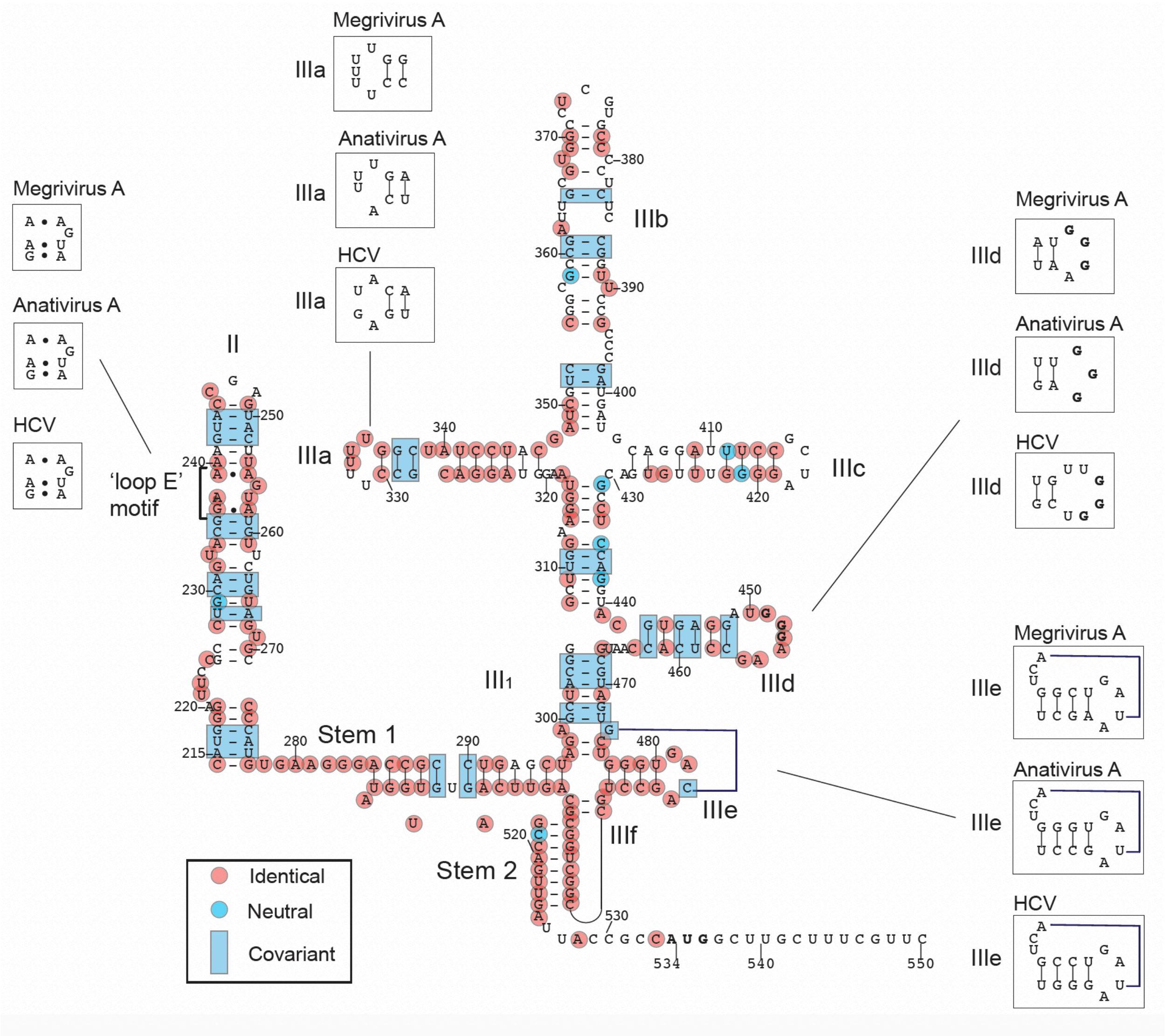

Supplementary Figure 2. Structural conservation between the type 4 IRESs of ruddy turnstone calicivirus and anativirus A1

Model of the RTCV IRES, marked to show sequence covariation between it and the type 4 IRES of Anativirus A1 isolate TW90A (NCBI Reference Sequence: NC_006553.1). Nucleotide identity and nucleotide differences that are structurally neutral (e.g. substitution of a G-U basepair by an A-U basepair) or compensatory (substitution of a G-C basepair by an A-U basepair) are as indicated in the inset key. Key sequence motifs in domains II and III are compared with analogous motifs in the type 4 IRESs of hepatitis C virus genotype 1 (NCBI Reference Sequence: NC_004102.1) ("HCV"), anativirus A1 isolate TW90A ("Anativirus A") and megrivirus A (NCBI Reference Sequence: NC_021201.1) ("Megrivirus A"). The apical G-rich motif in domain IIId is indicated by bold font.

```
                         10        20        30        40        50        60        70        80        90       100       110       120
                 ....|....|....|....|....|....|....|....|....|....|....|....|....|....|....|....|....|....|....|....|....|....|....|....|....|....
hwf182cal1  1075 ---AAHRPQAAPITAYQ-NPTLTDDQYREWQDARRHSGRNYTVDEYLLAKAALD-GLQRDVAANVQPLVDFLRDR-----------QTGLARMGQLQ-------------------------------- 1155
xftoti      1047 ------SNSRGMVRNFQAGVALSDEEYNTWRDYNTRVDSRATVNDFIAARESLA-NNSAITTERVAQLARWLQAR-----------NPNVL------EAQ----------------------------- 1122
Avocet      990  ------TGGRGQVRNYQAGVALSDQEYDTWREYNTRVDARATVNDFIAARESLA-RNAPITSERVSQLARWLQAR-----------APNDL------EPQ----------------------------- 1065
Pink-eared  1049 ----AGPNPRGMVRNYQAGIALSDEEYNTWREYNTRVDPRATVSDFITARDSLL-NNQVIATERIASLARWLQAR-----------NSPNL---GVFEAQ----------------------------- 1129
Crane       1056 ------SNQRGMVRNYQAGVALSDEEYNTWREYSTRVDRSATVSDFIAARDALA-NNTAIAQDRIQALARWLQSR-----------NNLL----GTFQ------------------------------- 1131
Nacovirus   982  ------AAGRGMVRNYQAGVALSDEEYNTWREYQSRVDRSATISDFIAARDALA-NNQAIAQERVAALATWMRAR-----------AAPEF------Q------------------------------- 1055
Wilkes      1013 ------SATRGLVRNYQAGVALSDDEYNSWREYNTRVDRSATVNDFIRAREMLQ-GIQATSTERVAALARWLRAR-----------DENVL------EPQ----------------------------- 1088
Duck        1213 ----TAPAPRGSIRQYR-GVALSDSEYETWQTYRATYDNTITAEEFIQARDAWR-GNQTVTNARLRSLTDWLRTR-----------EVGLDASGAVLQ------------------------------- 1293
Sanovirus   1033 ----NPPIPRGAIRQYR-GVALTDGEYETWQEYRKSYDPTITADDFVAARDAWK-GATTTLNTRLRTLADWLRTR-----------SSPVDASGAVLQ------------------------------- 1113
Grey_teal   1077 ----------SNTRRAYTGIALPDDQYNEWQTYRNRVDNTITVEDYVQAREALA-VERPVLEARIQALNRWMRAQ---------RARPTLVDSEAVLQ------------------------------- 1154
RTCV        1080 APPQHLPATPANVRRDFRGVALTDSEYSDWREYRTNIDPNVSVNDYIEAREYVR-GQLRNISPRLRDLAEHVKLK-----------SESEG--TGILQ------------------------------- 1163
Sapovirus   935  -AKGKNKRGRGA----RRAIALRDDEYDEWQDIIRDWRKEMTVQQFLDLKERAL-SGASDPDSQRYNA--WLELRAKRLSAGAYQHAVVDIIKSGHRREVIRTQVMRAP-R--------EPKGDTYDSE 1047
Vesivirus   961  -AKGKTKLKIGTYRGR--GVALTDDEYDEWREHNASRKLDLSVEDFLMLRHRAALGADDNDAVKFRS---WWNSRT-KMAND--YEDVTVIGKGGVKHEKI---------RTNTLKAVDRGYDVSFAEE 1071
Norovirus   871  GKKGKNKKGRGRPGVFR-TRGLTDEEYDEFKKRRESRGGKYSIDDYLADREREEELLERDEEEAIFGDGFGLKAT--RRSRKAERAKLGLVSGGDIRARKPIDWNVVGPSWADDDRQVDYGEKINFE-- 994
                                          *
                                         α1                  α2                  α3
```

Supplementary Figure 3. Calicivirus VPg sequences.

Calicivirus VPg sequences from representative members (name followed by GenBank accession number) of the genera Nacovirus (turkey calicivirus; JQ347522.1), Norovirus (murine norovirus; AY228235.2), Sanovirus (goose calicivirus H146; KY399947.1), Sapovirus (porcine sapovirus; KT922087.1) and Vesivirus (feline calicivirus; M86379.1) and from the IRES-containing sequences of Caliciviridae sp. isolate hwf182cal1 (MT138020.1), Caliciviridae sp. isolate xftoti59cal1 (MT138028.1, avocet calicivirus isolate MW21 (MH453804.1), pink-eared duck calicivirus I isolate MW23 (MK204416.1), Wilkes virus isolate Antarctic11 (MT025075.1), Caliciviridae sp. isolate yc-13 (KY312552.1) ("Crane"), duck calicivirus MW20 (MH453811.1), RTCV (ruddy turnstone calicivirus; MH453861.1) and grey teal calicivirus isolate MW09 (MK204392.1)), aligned using MAFFT (https://www.ebi.ac.uk/Tools/msa/mafft/). Conserved amino acid residues are highlighted in red, α-helices are indicated by open blue boxes and labeled, the invariant Tyrosyl residue that is linked to the viral RNA is indicated by an asterisk, and the C-terminal element in the murine norovirus VPg that binds eIF4G is highlighted in light blue.
